# Filtration Improves the Performance of a High-Throughput Screen for Anti-Mycobacterial Compounds

**DOI:** 10.1371/journal.pone.0096348

**Published:** 2014-05-02

**Authors:** Nancy Cheng, Melissa A. Porter, Lloyd W. Frick, Yvonne Nguyen, Jennifer D. Hayden, Ellen F. Young, Miriam S. Braunstein, Emily A. Hull-Ryde, William P. Janzen

**Affiliations:** 1 Center for Integrative Chemical Biology and Drug Discovery, Eshelman School of Pharmacy, University of North Carolina, Chapel Hill, North Carolina, United States of America; 2 Department of Microbiology and Immunology, University of North Carolina, Chapel Hill, North Carolina, United States of America; 3 Cancer Genetics Program, Lineberger Comprehensive Cancer Center, University of North Carolina, Chapel Hill, North Carolina, United States of America; 4 DMPK Advisors, Chapel Hill, North Carolina, United States of America; University of Padova, Medical School, Italy

## Abstract

The tendency for mycobacteria to aggregate poses a challenge for their use in microplate based assays. Good dispersions have been difficult to achieve in high-throughput screening (HTS) assays used in the search for novel antibacterial drugs to treat tuberculosis and other related diseases. Here we describe a method using filtration to overcome the problem of variability resulting from aggregation of mycobacteria. This method consistently yielded higher reproducibility and lower variability than conventional methods, such as settling under gravity and vortexing.

## Introduction

Tuberculosis (TB) is causing a global pandemic. Approximately 2 billion people are infected and nearly 9 million develop active disease each year [1]. India bears the heaviest burden, as 40% of the population is infected [1]–[5]. Since resistance of TB to existing drugs is becoming a serious problem, new therapies are urgently needed [6].

Traditional assays and models for screening anti-*Mycobacterium tuberculosis* compounds are lengthy and not well adapted to HTS [7]. Despite this, Ananthan, et al. [8] have successfully screened over 100,000 compounds for activity against *M. tuberculosis* in a 384-well plate assay, using gravitational settling of the supernatant to reduce variability.

Recently, the faster growing and safer relative of *M. tuberculosis*, *M. smegmatis*, was used to assay several thousand compounds [9]. The success of this effort led to the discovery and approval of bedaquiline, the first novel anti-TB compound in forty years [3]. Therefore, it is not surprising that as part of the search for new medicines, libraries of compounds are being screened with high-throughput methods for activity against *M. tuberculosis* and surrogate mycobacteria [10]. In addition to the successful discovery of bedaquiline, whole cell screens with mycobacteria have resulted in the nitroimidazole clinical trial candidates PA-824 [11] and OPQ-67683 (Delamanid), [12]and the diamine candidate SQ-109 [13] However, the tendency for mycobacteria to aggregate makes it difficult to distribute equal number of cells in each well of a microplate, leading to increased assay variability and lower probability of uncovering potential screening hits unless the cultures are manipulated to disaggregate the cells.

The hydrophobic and waxy mycolic acid layer in the cell wall is the cause of aggregation in mycobacteria [14], [15]. Without dispersing the bacteria to single cells, it is difficult to achieve accurate cell counts and equal distribution into microplate wells. Dispersants such as Tween 80 or Tyloxapol are commonly added to the culture media, and this partially mitigates bacterial ‘clumping ’[16]. Mechanical methods of disaggregation include repeated vortexing [17], sonication [10], needle dispersion [18], and gravitational settling of larger particles to form a supernatant [8]. Our attempts to use these mechanical methods in high throughput were not satisfactory. Either significant amounts of bacteria were lost, or the methods were overly cumbersome, time consuming, and only moderately effective.

Besides chemical and physical dissociation of the bacteria to single cells, others have used metabolic processes to enumerate Mycobacteria, such as incorporation of ^14^C palmitate [19] and the reduction of Alamar Blue (resazurin) [20]. These procedures have been validated by correlating their signals to the gold standard of counting colony-forming units (CFUs) on an agar plate. However, if the CFUs were derived from clumpy cultures, the correlation with the accurate radiometric or the fluorometric methods may be poor. Recently, some have suggested PCR as a way to enumerate the bacteria, but this method does not adequately differentiate between dead and living cells [21].

In this paper, we describe a method wherein a syringe fitted with a 5-µm filter is used to easily and rapidly disperse the clumps of *M. smegmatis* to a uniform, single-cell culture. This preparation provided high yields of homogenous single-cell suspensions in a matter of minutes. We then used a resazurin-resorufin microtiter plate assay method to quantify live bacteria in the sample. Filtering greatly reduced assay variability and, when applied to a high-throughput screen, was reproducible and precise.

## Materials and Methods

*M. smegmatis* strain mc^2^155 was obtained from ATCC. The bacteria were inoculated into Middlebrook 7H9 broth (BD Difco #271310) with 0.5% glycerol (Fisher Scientific BP229-4), 0.2% glucose (Sigma-Aldrich G8270), and 0.1% Tyloxapol (Sigma-Aldrich T8761). Frozen stocks from the same culture were used to reduce variability. Glycerol (15%) was added to cultures of *M. smegmatis* to reach an optical density at 600 nm (OD_600_ = 1) of one. Aliquots of 1-ml cultures were stored frozen at −80°C. As needed, a working solution of bacteria was made by adding 4 mls of 7H9 (supplemented as above) to 1 ml of a frozen stock. This working solution was also used for enumerating CFU of *M. smegmatis* on plates of 7H9 with agar (Fisher A360). To filter the cultures, 5-µm pore filters (Millipore SLSVO25LS) were used. The bacteria working solution was drawn into a syringe and expelled through the filter. Bac Titer-Glo, a cell viability reagent that measures intracellular ATP concentration, was obtained from Promega (G8230).

To assess our methodology of dissociating clumps, bacteria were either untreated, vortexed for three minutes at the highest setting, or filtered as described above. Cells were then diluted to 2.2×10^5^ CFU/ml and 45 µl added to each well of 384-well black assay plates with clear bottoms (Corning #3712) using a MultiDrop Combi (Thermo Fisher 5840400). Lids were attached and the plates were sealed in a zip-lock bag and placed into a 37°C incubator. Following a 24 or 48-hour incubation, 5 µl of 0.025% resazurin (Sigma-Aldrich, R7017) was added to each well. After 3–4 hours of incubation at 37°C, the fluorescence was measured by excitation at 530 nm and emission at 590 nm using a fluorimeter (EnSpire Alpha, Perkin Elmer). No major differences were observed between 24 and 48-hour incubation, therefore, as a more expedient method, we chose the overnight incubation procedure. To perform HTS, compounds were dispensed using a NanoScreen liquid handler (NanoScreen NSX 15360). The robot transferred 5 µl of 10 µMcompounds from 384-well compound plates (Greiner Bio One, 781280-1B) into 384-well Corning black assay plates, mentioned above. The Library of Pharmacologically Active Compounds (LOPAC), obtained from Sigma-Aldrich (LO1280), was used to validate this protocol.

*M. tuberculosis* H37Rv was grown in 5 ml 7H9 broth supplemented with 0.5% glycerol, 0.5% bovine serum albumin, 0.2% dextrose, 0.85% NaCl, and 0.05% Tyloxapol for 3 days at 37°C. Two ml of culture was removed and syringe-filtered through a 5-µm pore filter. An equal volume of 10% formalin was added to both the filtered and unfiltered bacteria and incubated at room temperature for one hour before removal from the BSL3 for microscopy using a Leica DMIRB Inverted Fluorescence/DIC Microscope with Photometric HQ2 camera.

## Results and Discussion

**Figure 1:**
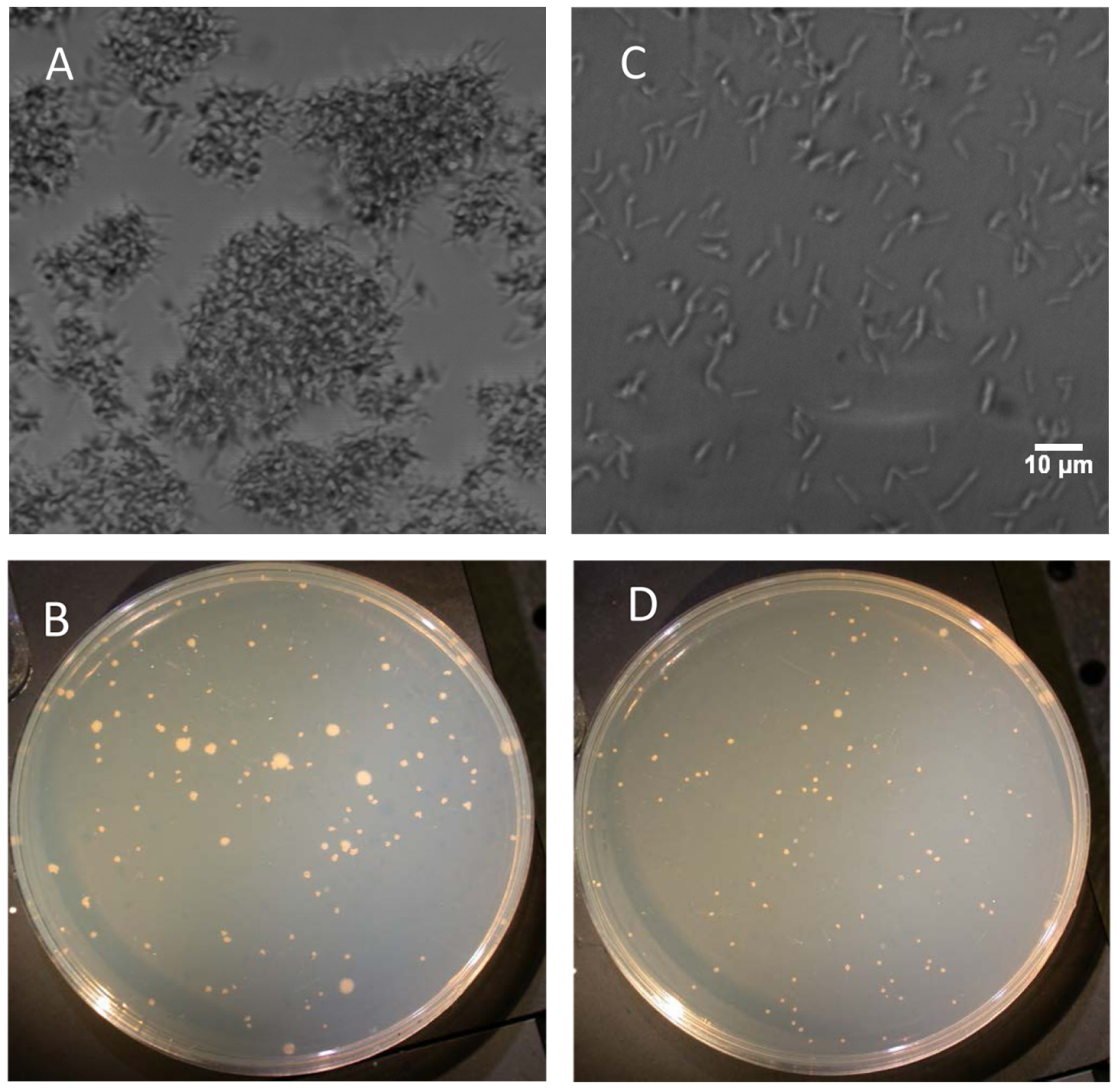
Comparison of unfiltered and filtered *M. smegmatis*. Unfiltered *M. smegmatis* under 40x magnification (A) and plated onto agar (B); *M. smegmatis* filtered through 5-µm pore filter under 40 x magnification (C) and plated onto agar (D), scale bar applies to both A and C.

We first used light microscopy to study the propensity of mycobacteria in culture to form clumps of thousands of individual organisms. These clumps are readily detected, as seen in Figure 1A. The aggregates vary widely in size, with large ones approaching 100 µm in diameter. When the *M. smegmatis* cultures were plated in the CFU-assay, colonies of different sizes were evident (Fig.1B), most likely as a result of different sized clumps of bacteria forming the colony. That is, a large colony came from a large clump of bacteria while a smaller clump formed a smaller colony. The lack of homogeneity in mycobacteria cultures made it difficult to obtain accurate and consistent CFU counts. To dissociate the clumps to achieve homogeneity in the culture, we vortexed the culture for 3 minutes at maximum speed; but the clumps remained in the culture (data not shown). Filtration, on the other hand, readily eliminated the clumps, leaving behind a uniform culture of single bacteria about 3–5 µm long (Fig. 1C). When the filtered cultures were plated, the resulting colonies were uniform in size (Fig. 1D).

One drawback of other methods currently used to produce single-cell suspensions of *Mycobacterium* spp. is the loss of a significant fraction of the cells. This problem is particularly severe with the gravitational settling method [8]. After settling at 1 g for 1 hour, a mostly single-cell suspension is obtained by utilizing the upper half of the culture, but at the cost of a loss of 70% of the cells in the culture. At first it appeared that our filtration method also resulted in a significant loss of cells. The filtered cultures had an OD_600_ of about one-third of the unfiltered sample, OD_600_ = 0.119 and OD_600_ = 0.342 respectively (Table 1) suggesting that 66% of bacteria had been lost during filtration. However, when we either measured the ATP level in the culture with a luminescence assay, or enumerated the bacteria by plating them on agar, we did not observe this loss (Table 1). We believe that the OD readings decrease because OD_600_ is dependent on light-scattering, not absorbance [22]–[24]. Light is scattered as the sixth power of the radius of a particle, so the larger particles in the unfiltered cultures were disproportionately represented in the OD_600_ signal [25], artificially increasing the signal.

**Table 1:** Recovery of *M. smegmatis* after Vortexing and Filtration.

	Unfiltered	Vortexed	Filtered
OD at 600 nM	0.342±0.010	0.317±0.009	0.119±0.004
Bac Titer-Glo	220,000±10,243	224,000±8,539	189,000±5,066
CFU per 0.0007 µl	38.75±18.26	61.0±32.99	47.75±9.29

Mean ±Standard Deviation; n = 4.

**Figure 2:**
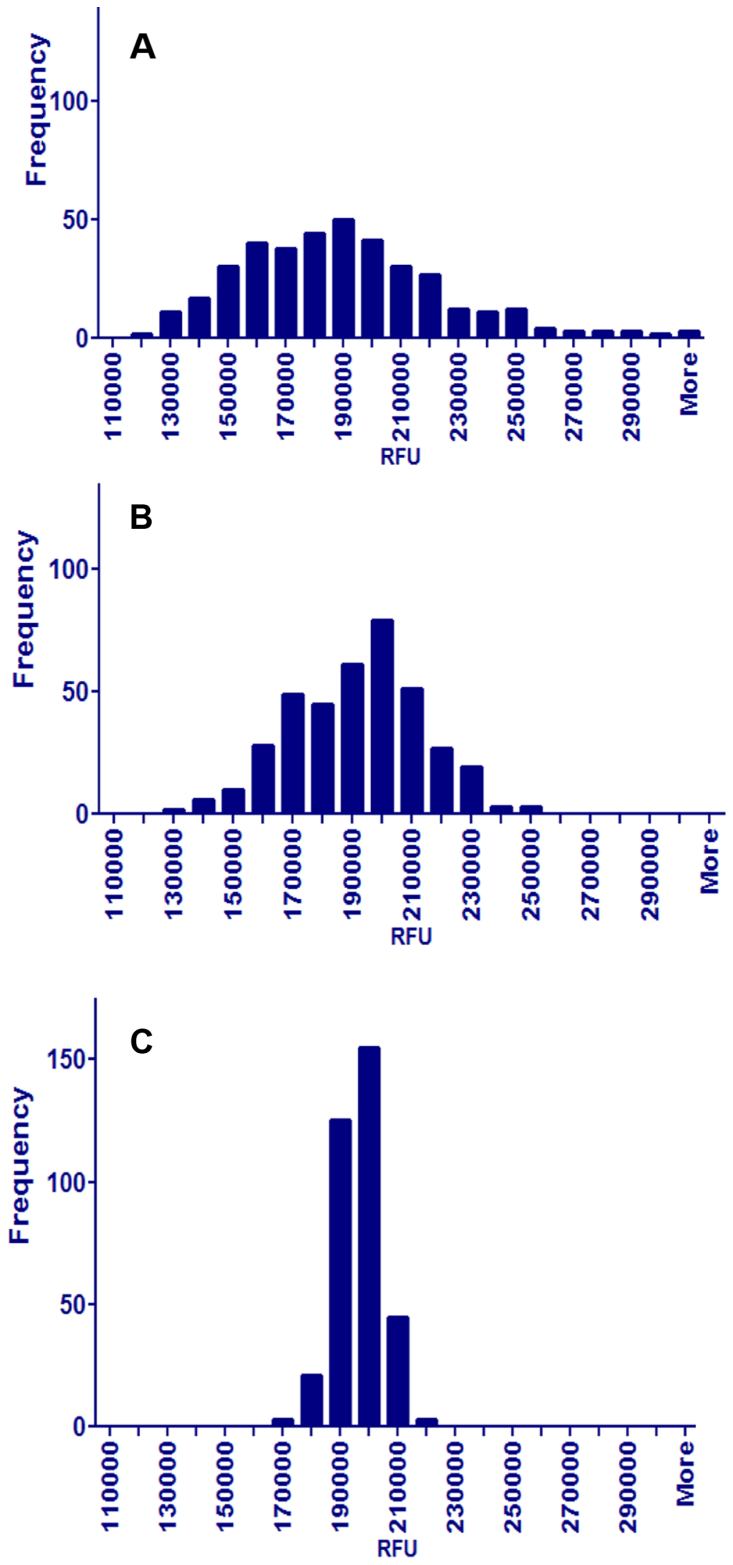
Histogram of unfiltered, vortexed and filtered *M. smegmatis*. Distributions of resorufin fluorescence signals from 384 wells of a 384-well plate contained unfiltered (A), vortexed (B) or filtered (C) *M. smegmatis*. After the treatment, the bacteria were distributed into the 384-well plates followed by the addition of resazurin, which was converted to resorufin by the living bacteria.

Our high throughput screening strategy employs cultures in 384-well plates incubated with resazurin to assess viability [18], [26], [27]. To precisely measure inhibition in the presence of compounds, we need to ensure that equal numbers of cells are dispensed into each well. We compared the repeatability of dispensing either unfiltered, vortexed, or filtered cultures. Histograms of these data revealed that samples of the unfiltered cultures were highly variable, with a broad ‘tail’ of many wells having large fluorescence and a non-normal, bi-modal distribution with a coefficient of variation (CV) greater than 28%, (Fig 2A). Samples from cultures that had been vortexed were less variable, with a peak of fluorescence at about 200,000 units, but the distribution was still non-normal and bi-modal with a CV greater than 22% (Fig 2B). In contrast, samples from filtered cultures were normally distributed with a CV of about 7% (Fig 2C). These differences were observed in five separate experiments.

**Figure 3:**
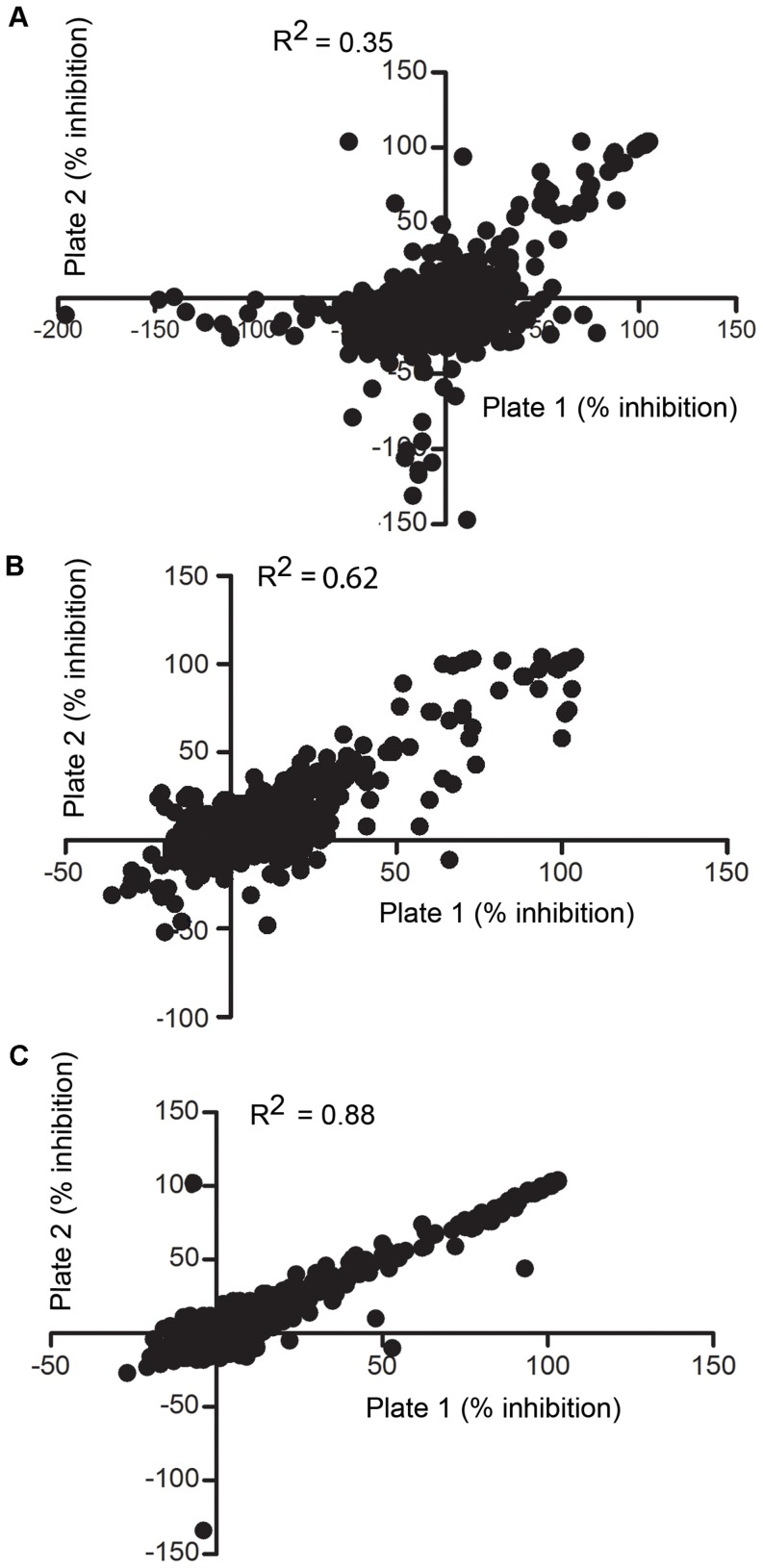
Agreement analysis of duplicate plates from unfiltered, vortexed and filtered bacteria. Correlation of two duplicate assay plates tested against LOPAC compounds using unfiltered bacteria (A), vortexed bacteria (B) and filtered bacteria (C).

To test if filtration improved the performance of HTS of compounds against *M. smegmatis*, we performed replicate assays of a diverse set of compounds and compared the results [28]. We dispensed populations of unfiltered and filtered bacteria into duplicate 384-well plates that contained compounds from the LOPAC library. A pivot plot of the percent inhibition in the first replicate plate compared to the inhibition in the second plate is shown for the unfiltered (Fig. 3A), vortexed (Fig. 3B) and filtered (Fig. 3C) bacteria. The correlation between the replicate assays is excellent with the filtered cultures, indicating that the assay is repeatable. The Z’ score [29] calculated from the results with filtered cells is greater than 0.9 while unfiltered and vortexed cultures have values of 0.35 and 0.62 respectively. The high Z’ values with filtered cultures (Fig. 3C) indicate that this method will give better HTS data than unfiltered and vortexed cultures that have lower Z’ values and higher standard deviations (Fig. 3A and 3B). Compared to untreated cultures, vortexing did improve the Z’ and the standard deviation, but not as much as filtering the cultures.

**Figure 4:**
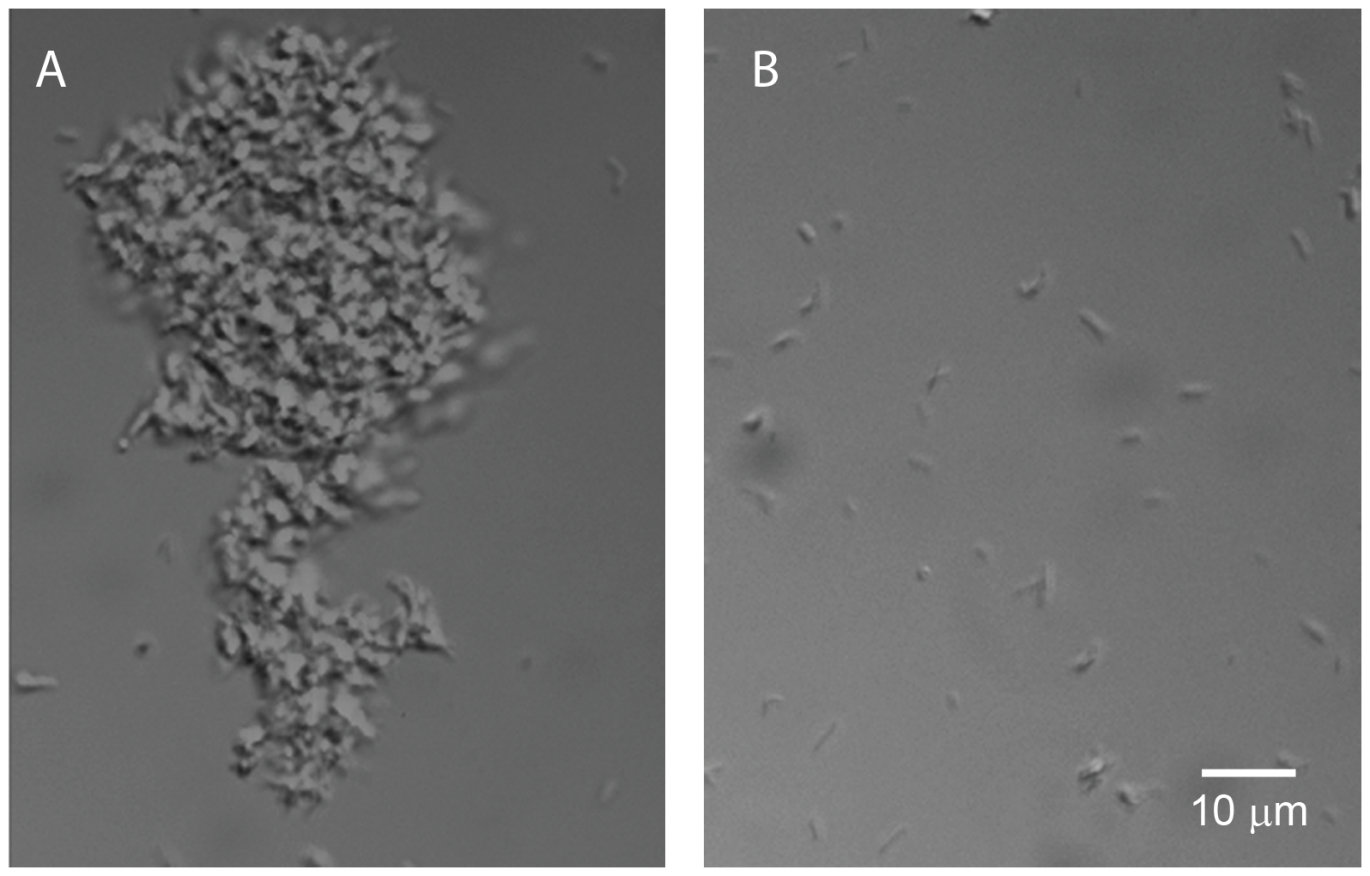
Micrograph of unfiltered and filtered *M. tuberculosis.* Unfiltered *M. tuberculosis* under 40x magnification (A) and filtered through 5-µm pore filter under 40x magnification (B), scale bar applies to both A and B.

When this simple filtration method was extended to *M. tuberculosis*, we observed similar results, i.e. single cells were produced from clumps in this species as well (Fig. 4A and 4B). Therefore this technique for the production of single cells from aggregates is not limited to the model species *M. smegmatis*.

Filtration does not appear to impact the growth or metabolism of the mycobacteria because filtered and unfiltered bacteria have similar growth rates and ATP levels. In any case, the filtered cultures reform clumps within a few hours (data not shown). This method meets the criteria required for quality data from HTS [28], [30], and has quickly become our standard technique. We used log-phase cultures in our screen of the LOPAC library to provide greater sensitivity to compound inhibition. It is possible that a different hit profile would emerge if the cultures were allowed to reach stationary phase prior to the addition of test compounds, but filtered cultures would still be desirable in this situation, since most of the variability in plated based assay occurs at the time that the cells are dispensed into the wells. Besides filtering bacteria in different growth phases, we have also filtered *M. smegmatis* cultured in media with succinate as the sole carbon source. We observed the same degree of aggregation with the succinate media (data not shown), and after filtration we again obtained single-cell suspensions. We recommend that this filtration procedure be incorporated into any protocol where mycobacteria are assayed in a microplate format.
